# Peri-operative chemotherapy in the management of resectable colorectal cancer pulmonary metastases

**DOI:** 10.1186/1471-2407-12-326

**Published:** 2012-08-01

**Authors:** Eliza A Hawkes, George Ladas, David Cunningham, Andrew G Nicholson, Katharina Wassilew, Yolanda Barbachano, Gihan Ratnayake, Sheela Rao, Ian Chau

**Affiliations:** 1 Department of Medicine, Royal Marsden Hospital, Downs Road, Sutton, Surrey, UK SM2 5PT; 2 Department of Surgery, Royal Brompton and Harefield NHS Foundation Trust, London, UK; 3 Department of Histopathology, Royal Brompton and Harefield NHS Foundation Trust, and National Heart and Lung Division, Imperial College, London, UK; 4 Department of Clinical Research and Development, Royal Marsden Hospital, London and Surrey, UK

**Keywords:** Colorectal cancer, lung resection, peri-operative chemotherapy

## Abstract

**Background:**

Surgery is often advocated in patients with resectable pulmonary metastases from colorectal cancer (CRC). Our study aims to evaluate peri-operative chemotherapy in patients with metastastic CRC undergoing pulmonary metastasectomy.

**Methods:**

Patients treated for CRC who underwent pulmonary metastasectomy by a single surgeon were identified. Outcome measures included survival, peri-operative complications, radiological and histological evidence of chemotherapy-induced lung toxicities.

**Results:**

Between 1997 and 2009, 51 eligible patients were identified undergoing a total of 72 pulmonary resections. Thirty-eight patients received peri-operative chemotherapy, of whom 9 received an additional biological agent. Five-year overall survival rate was 72% in the whole cohort - 74% and 68% in those who received peri-operative chemotherapy (CS) and those who underwent surgery alone (S) respectively. Five-year relapse free survival rate was 31% in the whole cohort - 38% and ≤18% in CS and S groups respectively. Only 8% had disease progression during neoadjuvant chemotherapy. There were no post-operative deaths. Surgical complications occurred in only 4% of patients who received pre-operative chemotherapy. There was neither radiological nor histological evidence of lung toxicity in resected surgical specimens.

**Conclusions:**

Peri-operative chemotherapy can be safely delivered to CRC patients undergoing pulmonary metastasectomy. Survival in this selected group of patients was favourable.

## Background

Colorectal cancer (CRC) accounts for about 10% of all cancers worldwide with 1.2 million new cases and over 600,000 deaths in 2008 [1]. Despite a lack of randomised, prospective data, resection of both hepatic and pulmonary metastases has become a standard of care for selected CRC patients [2]. While not all patients benefit from surgery, a recent systematic review found 5-year survival after complete resection of lung metastases ranged between 40-68% [3] – higher than expected from use of modern combination chemotherapy in unresected metastatic CRC [4].

Numerous published series of surgical outcomes have identified negative prognostic factors including presence of multiple (i.e. >2) or bilateral metastases [5,6], elevated pre-thoracotomy carcinoembryonic antigen (CEA) level (>5 ng/mL) [7], largest lesion > 30 mm [8], local nodal involvement [9,10], and higher stage of primary tumour [6] yet these have not routinely been adopted into the clinical decision-making model due to the heterogeneous and contradicting nature of the published literature.

Peri-operative chemotherapy has been incorporated into the clinical management of resectable liver metastases following the encouraging results of the European Organisation for Research and Treatment of Cancer (EORTC) 40983 study using peri-operative chemotherapy [11]. Targeted agents such as bevacizumab and cetuximab are also utilised for selected patients with liver-only metastatic disease [12-14]. Despite advances made in peri-operative treatment of resectable hepatic metastases, there remains a relative paucity of data regarding its role in pulmonary metastasectomy, and any resulting toxicity. Previously we reported on 11 patients from our joint-institution (Royal Marsden/Royal Brompton Hospitals) series [15]. In this current study, we report on an expanded cohort treated until March 2009 as well as evaluating the role of pre-operative positive emission tomography (PET) and examining for evidence of chemotherapy-induced pulmonary toxicity.

## Methods

The study was approved by our Institutional Review Board (CCR SE3343). Patients ≥18 years of age with a histological diagnosis of colorectal adenocarcinoma who underwent a curative-intent pulmonary metastasectomy were deemed eligible. Patients undergoing oncological treatment at the Royal Marsden Hospital (RMH) were identified from a list of pulmonary metastases resections performed at the Royal Brompton Hospital (RBH) by a single surgeon (GL) between January 1997 and March 2009. All information was collated from patient records at both RMH and RBH. Based on the RMH GI unit guidelines, CRC patients were reviewed in a multidisciplinary meeting, and selected for perioperative chemotherapy if disease-free interval <2 years and the nature of the pulmonary nodules was deemed to be malignant. Data were collected on age, gender, primary tumour site and date of primary tumour resection, adjuvant therapy related to the primary tumour, date of lung metastases diagnosis, disease-free interval (DFI) (calculated from the date of primary colorectal tumour resection to date of diagnosis of first lung metastasis), pulmonary surgery, relapse site and date of death or last follow up. Details of pulmonary surgery, peri-operative chemotherapy related to pulmonary metastases, including response, toxicities and complications, as well as pre-operative CEA levels were recorded. Following pulmonary resection, standard follow-up at this institution consisted of 3-monthly clinical review and CEA levels for the first year then 6-monthly reviews plus CEA levels thereafter as well as 6–12 monthly imaging with computed tomography (CT) scan.

Patients were selected for pulmonary metastasectomy based on the following criteria: 1) primary tumour controlled; 2) the only identifiable sites of metastatic disease being lung or liver or both; 3) disease stability in terms of number of lesions confirmed on interval scans; 4) complete resection of all deposits feasible and 5) patient fit for planned procedure [2]. Details of surgical approach are outlined in Appendix A.

All those undergoing neoadjuvant chemotherapy underwent a baseline and pre-operative CT scan. However 2 patients had scans externally with no report available. A documented reduction in size of lesions was recorded. Standard radiological response criteria such as RECIST [16] were not possible to use due to small size (often sub-centimetre) of pulmonary metastases.

Haematological and biochemical toxicities were graded according to the National Cancer Institute Common Terminology Criteria for Adverse Events version 3.0 (CTCAEv3.0). Clinical toxicities were also graded, based on documented findings and the treatment required. Evidence of lung toxicities such as pneumonitis and pulmonary fibrosis, as documented on CT scan, was also recorded.

Resected paraffin-embedded lung tissue specimens were examined by two specialised pulmonary pathologists (AGN and KW), who were blinded to all clinical information concerning chemotherapy. Samples were assessed for viable tumour. Non-tumour lung tissue from each specimen was also assessed for evidence of chemotherapy-related toxicity including pulmonary eosinophilia, interstitial fibrosis and/or inflammation unrelated to the locale of the tumour. These findings were subsequently correlated with clinical data to evaluate the possible role of neoadjuvant chemotherapy in lung toxicity.

### Surgical approach

The main aim was complete resection of all deposits with clear margins, whilst preserving as much lung parenchyma as possible. Pre-operative biopsy of suspect lesions was not performed. All lesions visible on preoperative CT scan were recorded and mapped preoperatively. Lesions which had achieved a complete radiological response from neoadjuvant chemotherapy and were no longer visible were not resected. Muscle-sparing, limited postero-lateral thoracotomy was the most utilised approach. Bilateral disease was resected by staged bilateral thoracotomies 4-6 weeks apart, or rarely by median sternotomy and synchronous bilateral resection. The use of double-lumen endotracheal tubes allowed single lung anaesthesia, with the operated lung being collapsed during the procedure. Following removal of all known lesions, palpation was used to identify additional lesions not detected on CT scan. In our experience, on average 25% more nodules were discovered this way, hence Video Assisted Thoracoscopy is rarely used in our metastasectomy practice. A wide range of resection techniques were used. Small nodules were removed by “precision resection”, using diathermy spatula or laser beam, with a sphere of surrounding healthy lung parenchyma to ensure clearance. Deeper parenchymal lesions, underwent localised resection via an anatomical sub-segmental to ligate individual feeding vessels and bronchi. Sizeable tumours involving larger hilar structures were removed using anatomical segmentectomy or lobectomy, always in combination with nodal dissection at N1 level, whilst pneumonectomy was only rarely justified. A combination of various techniques is often used in the same patient when multiple deposits are present.

Since a lobectomy with a systematic nodal dissection is the accepted minimum resection for primary lung cancer, distinguishing a new lung primary adenocarcinoma from a solitary colonic metastasis was crucial, in planning the appropriate resection. Given that up to 80% of primary lung adenocarcinomas stain positive for Thyroid Transcription Factor -1 (TTF-1), we regularly used intraoperative immunohistochemistry for TTF-1 on frozen section specimens to make this distinction. In February 2010 we started using a 1318 nm lung laser device, and laser assisted lung resection was now routinely used in all metastasectomy patients in our practice.

### Statistical considerations

The primary endpoint of the study was overall survival (OS). Secondary endpoints include relapse-free survival (RFS), response to neoadjuvant chemotherapy where applicable and toxicity. Overall survival was measured from date of first treatment for the pulmonary metastases (chemotherapy or surgery) to death from any cause and censored at last follow-up. RFS was measured from date of initial pulmonary surgery to date of relapse, death from any cause or date of last follow-up. RFS and OS were estimated using the Kaplan Meier method [17].

Univariate log-rank analyses were performed to assess the impact of clinico-pathological factors on survival. Multivariate Cox regression analysis was only performed if >1 factor were found to be significant (p < 0.05). Factors included were elevated pre-treatment CEA (>institutional upper limit of normal), number of pulmonary metastases (solitary versus multiple), largest metastasis (≥30 mm versus <30 mm), involvement of resected thoracic lymph nodes, disease-free interval (<12 months versus ≥12 months), PET avidity of lung lesions (positive versus negative) and peri-operative chemotherapy (peri-operative cytotoxic drugs versus cytotoxic drugs plus a targeted agent versus surgery alone). No formal statistical comparison of toxicities or complications was performed due to small numbers of events.

All analyses were performed using SPSS (PASW) version 18.

## Results

Fifty-one eligible patients underwent 72 curative resections for pulmonary metastases. Table 1 shows the baseline characteristics of eligible patients. Of 51 patients in our cohort, 38 (75%) received neoadjuvant and/or adjuvant chemotherapy relating to their pulmonary resection. In those with no metastatic disease at diagnosis, the median DFI was 24.1 months (range 2.8 to 64.1) for the whole cohort - 20.5 months (range 11.7 to 64.1) in the peri-operative chemotherapy group (CS) and 27.5 months (range 2.8 to 52.8) in the surgery-alone group (S). However, 11/38 (29%) patients in the CS group presented with synchronous metastases at the time of diagnosis.

**Table 1:** Patient characteristics

**Patient characteristics**	**Patients who received peri-operative chemotherapy**	**Patients who underwent surgery alone**	**Total cohort**
**Number of patients**	38	13	51
**Number of pulmonary resections**	56	16	72
**Age median (range)**	61.4 (41–78)	65 (49–78)	63 (41–78)
**Male:female**	23:15	7:6	30:21
**Site of primary**			
Colon	20	7	27
Rectum	18	6	24
**Stage at diagnosis**			
Stage II	8	6	14
Stage III	20	4	24
Stage IV	11	3	14
Site of metastases			
Liver	2	3	5
Lung	8	0	8
Lung + Omentum	1	0	1
**Adjuvant chemotherapy for primary CRC**			
Yes	24	7	31
With neoadjuvant CRT	3	4	7
With neoadjuvant chemo + CRT	3	0	3
With Adjuvant RT	0	1	1
No	8	1	9
**Site of first metastases**			
Lung only	29	10	39
Lung + liver	2	0	2
Liver only	5	3	8
Peritoneum	1	0	1
Nodal (para-aortic)	1	0	1
CEA pre-treatment*			
Recorded	35 (49 resections)	10 (12 resections)	45 (61 resections)
Not Recorded	3 (7 resections)	3 (4 resections)	6 (11 resections)
>institutional ULN	7 (8 resections)	2 (2 resections)	9 (10 resections)
Median CEA (range)	2 μg/dl (1–1094)	1.5 μg/dl (<1-10)	2 μg/dl (<1-1094)

Numbers in table refer to number of patients unless otherwise specified.

*either prior to neoadjuvant chemotherapy or prior to surgery if no neoadjuvant chemotherapy given.

Forty-five patients had CEA levels performed pre-thoracotomy, with an elevated result in only 9 patients. ^18^FDG-PET was performed in 45 patients with only 8 PET scans demonstrating no FDG-avidity in the lung lesions. The timing of the PET scan did not affect FDG-avidity, even in those undergoing neoadjuvant chemotherapy. Table 2 shows the comparisons among CT, PET and subsequent histology. Concordance with number of confirmed pulmonary metastases diagnosed histologically was higher in PET than CT (72% versus 61%) for those with FDG-avid lesions. The median size of the largest lesion was higher in the PET positive group compared to PET negative group (15 mm versus 8 mm respectively).

**Table 2:** Concordance between radiology, PET and histology

	**PET positive**	**PET negative**	**No PET done**
	**n (%)**	**n (%)**	**n (%)**
Patients*	39 (81)	8 (18)	6 (12)
**Resections**	48 (86)	8 (14)	16 (22)
**Timing of pre-thoracotomy PET**			
Before neoadjuvant chemotherapy	16	3	-
After neoadjuvant chemotherapy	12	1	-
No neoadjuvant chemotherapy	20	4	-
**CT identified metastases**			
Median number (range)	1 (1–6)	2 (1–3)	2 (1–11)
**PET identified metastases**			
Median number FDG avid (range)	1(1–6)	-	-
**Histologically identified metastases**^**#**^			
Median number (range)	1 (1–6)	2 (1–4)	1 (1–12)
**Median size of largest nodule (range)**	15 mm (5–60)	8 mm (3–15)	15 mm (2–25)
**Concordance rates on number of metastases**^**+**^			
CT + PET	34 (71)	-	-
CT + Histology	28 (61)	4 (50)	10 (71)
PET + Histology	33 (72)	-	-
CT + PET + Histology	26 (57)	-	-

^*^2 patients had FDG-avid lung lesions prior to one lung resection but no FDG avid lung lesions prior to a subsequent lung resection.

^#^Histology obtained at time of resection. All lesions considered involved had viable tumour.

^+^histology not available on 1 PET positive patient, and 2 patients who did not have a PET.

### Chemotherapy

Of the 38 patients (75%) who underwent peri-operative systemic therapy, 36 received it with their initial resection, while 2 patients only received chemotherapy with subsequent resections. Table 3 shows details of peri-operative chemotherapy. Nine patients received targeted biological agents combined with neoadjuvant chemotherapy. All targeted biological treatments were administered in combination with an oxaliplatin or irinotecan-based chemotherapy doublet. Post-operative chemotherapy plus targeted therapy was given to all patients receiving bevacizumab and 1 patient who received neoadjuvant cetuximab. In this cohort of 38 patients, chemotherapy was delivered in a total of 49 resections; most commonly peri-operatively (n = 22 resections), with neoadjuvant alone given in 17 resections and adjuvant alone after 10 resections. Seven patients who underwent multiple pulmonary resections received no systemic peri-operative treatment for at least 1 of these resections. The median number of chemotherapy cycles administered was 4 (range 2–12) pre-operatively and 5 (range 2–12) post-operatively. Of 30 patients who received neoadjuvant chemotherapy, 8 (27%) developed treatment-related complications (9 events). Post-operative chemotherapy-related complications occurred more frequently, in 14 patients undergoing adjuvant chemotherapy (54%, 19 events).

**Table 3:** Details of systemic chemotherapy

	**Patients who received chemotherapy**
	**(n = 38 patients undergoing 56 resections)**
**Timing of treatment**	
**Chemotherapy plus targeted agent**	n = 11 resections (in 9 patients)
Neoadjuvant plus adjuvant	7 (64%)
Neoadjuvant alone	4 (36%)
Adjuvant alone	0
**Chemotherapy alone**	n = 38 resections (in 29 patients)
Neoadjuvant plus adjuvant	15 (39%)
Neoadjuvant alone	13 (34%)
Adjuvant alone	10 (26%)
None*	n = 7 resections
**Type of systemic treatment**	
**Targeted agents**	n = 11 resections (in 9 patients)
Bevacizumab	8
Cetuximab	2
Sunitinib	1
**Chemotherapy alone**	n = 38 resections (in 29 patients)
Oxaliplatin	22
Irinotecan	6
Mitomycin	5
5FU/Cape alone	5
**Number of cycles**	Median (range)
**Pre-operative**	4 (2–12)
Targeted agents	6 (4–12)
Chemo alone	4 (2–8)
**Post-operative**	5 (2–12)
Targeted agents	6 (4–12)
Chemo alone	4 (2–12)
**Chemotherapy-related complications**	
**Pre-operative**	9 events (8 patients)
G3/4 neutropenia	2
G3/4 Thrombocytopenia	1
≥G2 neuropathy	2
G3/4 diarrhoea	1
G3/4 Chest pain	2
G3 VTE (DVT)	1
**Post-operative**	19 events (14 patients)
G3/4 neutropenia	4
G3/4 Thrombocytopenia	1
≥G2 neuropathy	3
G3/4 diarrhoea	3
G3 fatigue	2
G3 infection	1
G3 GI bleed	1
G3 PPE	1
G3 stomatitis	1
G3 nausea	1
VTE (pulmonary embolism)	1

Numbers in table refer to number of resections unless otherwise specified.

*7 resections occurred where patients received no chemotherapy, but had received for a prior or subsequent resection.

*VTE *venous thromboembolism, *DVT *deep vein thrombosis.

None of the CT scans performed following chemotherapy demonstrated fibrotic changes or pneumonitis suggestive of chemotherapy-related lung toxicity. Metastatic colorectal adenocarcinoma was confirmed on independent histological review in all cases with no histological complete responses (ie tumour necrosis or fibrosis in the absence of viable tumour) seen. Histopathological evaluation also demonstrated no evidence of chemotherapy-related changes to the non-cancerous lung parenchyma in any cases.

### Surgical procedures

Table 4 shows surgery details including type of resection, metastases removed, lymph node dissection, time from last dose of neoadjuvant treatment to surgery and surgical complications. All pulmonary resections were R0 (complete macro- and microscopic resection). Twelve patients had bilateral sequential resections, with no chemotherapy administered between. Median time to surgery after last dose of neoadjuvant vascular endothelial growth factor (VEGF) inhibitor (bevacizumab or sunitinib) was 37 days (range 25–85), whereas for all other neoadjuvant treatment median time to surgery was 48.5 days (range 14–207); the 207 day delay was in a patient who had significant post-operative complications from original bowel surgery. The median number of metastasis removed was 2.5 (range 1–19). Median metastasis size was 15 mm (range 2 to 60 mm). Surgical complications were uncommon (Table 4). Only 4% of patients undergoing pre-operative chemotherapy experienced surgical complications. Twelve patients also underwent hepatic metastasectomy during the course of their treatment.

**Table 4:** Details of surgery and surgical complications

**Details of surgical resections**	**Peri-operative chemotherapy**	**Surgery alone**	**Total cohort**
	**n = 56 resections (38 patients)**	**n = 16 resections (13 patients)**	**n = 72 resections (51 patients)**
**Type of resection**			
Wedge	37	10	47
Segmental	8	2	10
Lobectomy	8	4	12
Pneumonectomy	1	0	1
Not recorded	2	0	2
**Metastases removed**			
Solitary	16	4	20
Multiple	38	12	50
Unknown	2	0	2
**Median size (range)**	14.5 mm (2–60)	15 mm (4–25)	15 mm (2–60)
**Lymph node dissection**			
Yes	22	5	27
**Positive histology**	4/22 (18%)	1/5 (20%)	5/27 (19%)
No	31	10	41
Unknown	3	1	4
**Time from last dose of neoadjuvant treatment to surgery**	Median days (range)		
Any treatment	48.5 (14–207)		
Chemotherapy +	37 (25–85)		
VEGF inhibitor			
Surgical Complications^*^	3/68 (4%)	5/16 (31%)	8/84 (9%)
Prolonged air leak	3	0	3
Haemorrhage^**^	0	1	1
CVA	0	1	1
Cardiac Arrhythmia (fast AF)	0	1	1
Small bowel ileus or obstruction	0	2	2

Numbers in table refer to number of resections unless otherwise specified.

^*^Surgical complications were evaluated in all operations performed, hence the bilateral sequential resections being counted as separate events and the total n = 86, not 72.

^**^The patient was fully anticoagulated.

*CRC *colorectal cancer, *CRT *chemoradiation, *RT *radiotherapy, *CEA * carcinoembryonic antigen, *ULN *upper limit of normal, *VEGF *vascular endothelial growth factor, *CVA *cerebrovascular Accident, *AF *atrial Fibrillation.

### Efficacy

**Figure 1:**
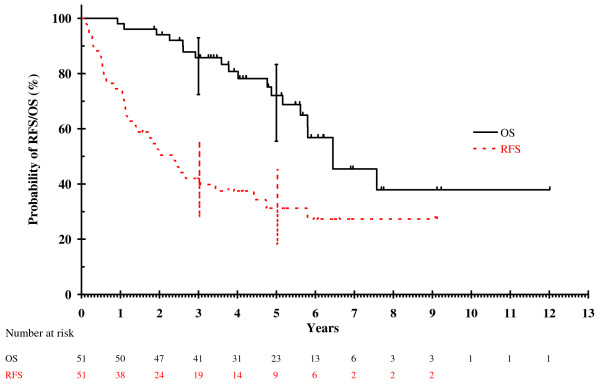
Overall and relapse free survival for whole cohort.

**Figure 2:**
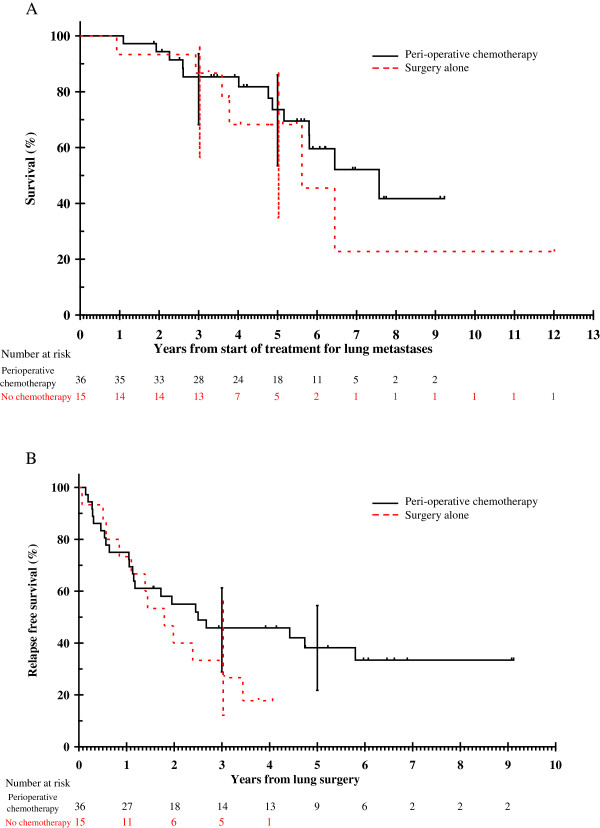
A Overall survival by treatment group; 2B Relapse free survival by treatment group.

**Figure 3:**
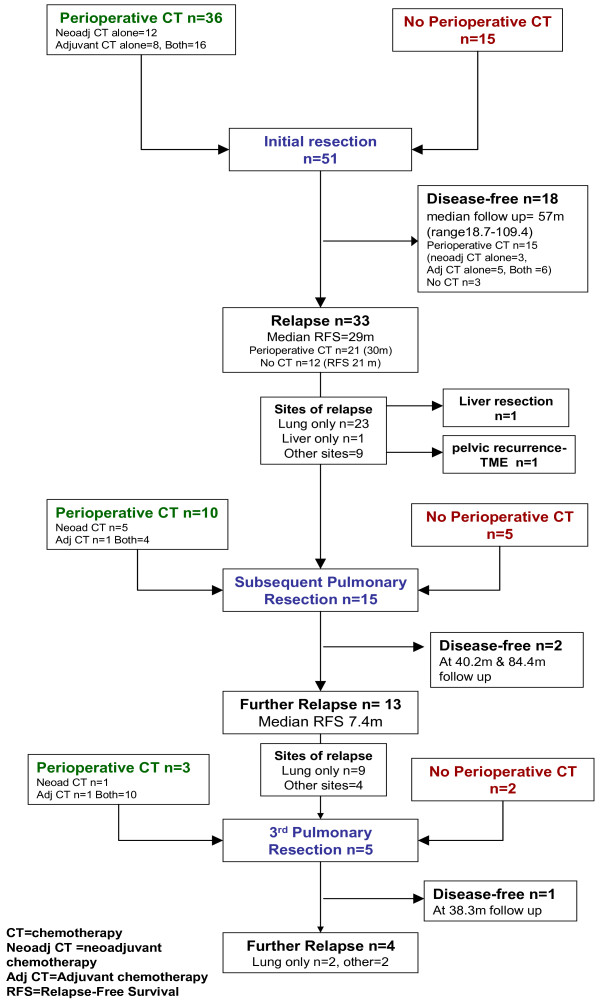
Outcomes of pulmonary resections.

Median follow up for the entire cohort was 60 months. The median OS in the entire cohort was 77 months (95% confidence interval [CI]: 55-99 months) and 5-year survival rate was 72% (95% CI: 55-83%). Eighteen patients remained disease free after initial resection. Five-year RFS for the entire cohort was 31% (95% CI: 18-45%). Figure 1 shows the OS and RFS for entire cohort. Five-year OS rates were 74% (95% CI: 53-86%) and 68% (95%: CI 35-87%) in CS and S groups respectively. Five-year RFS rates were 38% (95% CI: 22-54%) and ≤18% in CS and S groups respectively. Figures 2 and 3 show the OS and RFS respectively for the CS vs. S groups. A reduction in tumour size was seen in 21 patients (undergoing 23 of 39 resections - 62%) where neoadjuvant chemotherapy was administered. In a further 10 patients (11 resections or 30%), stable disease was seen on pre-thoractomy imaging after neoadjuvant treatment, with 3 incidents of disease progression. Thus, the disease control rate from neoadjuvant chemotherapy was 92%. Table 5 summarises the efficacy outcomes. Figure 3 shows details of relapse and further treatment.

**Table 5:** Summary of efficacy results

	**Total cohort**	**Peri-operative chemotherapy**	**Surgery alone**
	**n = 51**	**n = 38**	**n = 13**
**Number of patients**	51	38	13
**Disease-free interval (range)**	24.1 months	20.5 months	27.5 months
	(2.8-64.1)	(11.7-64.1)	(2.8-52.8)
**Overall survival**			
Median (95% CI)	77 months (55–99)	91 months (61–121)	67 months (39–96)
3-year (95% CI)	86% (72–93)	85% (68–93)	87% (56–96)
5-year (95% CI)	72% (55–83)	74% (54–86)	68% (35–87)
**Relapse free survival**			
Median (95% CI)	29 months (18–39)	30 months (0–68)	21 months (13–30)
3-year (95% CI)	42% (28–55)	46% (29–61)	38% (12–56)
5-year (95% CI)	31% (18–45)	33% (22–54)	≤18%^*^
**Response to neoadjuvant chemotherapy**			
		n = 39 resections	
Complete response		0	
Tumour reduction		23 (62%)	
Stable disease		11 (30%)	
Disease control		34 (92%)	
Tumour progression		3 (8%)	
No scans available		2	

^*^Not estimable as no events occurred at year 5 and only 1 patient left at risk at year 4 where RFS was estimated at 18%.

### Prognostic Factors

In an univariate analysis, only positive lymph node involvement was significant for survival (p = 0.022), however only 19 patients had lymph node resection at the time of pulmonary resection. All other factors assessed were non-significant and hence multivariate analysis was not performed.

With respect to RFS, no prognostic factors were found to be significant in univariate analyses. However, there was a trend towards improved RFS with chemotherapy alone compared to surgery alone or chemotherapy plus a targeted agent (p = 0.058).

## Discussion

This study focused on the effects of peri-operative chemotherapy in the setting of pulmonary resection for colorectal lung metastases. We believe this is the first dedicated published series on peri-operative chemotherapy. The absolute 5-year survival rate was higher in the CS group compared with S group (74% versus 68%) as well as 5-year RFS (38% versus ≤18%) despite a shorter DFI. Lymph node involvement was the only significant prognostic factor found in our cohort but only in univariate analysis. The numbers were too small to compare lymph node involvement between the perioperative chemo vs no chemo groups or to perform a multivariate analysis. Also, the more recent procedures almost routinely involved lymph node resection whereas earlier procedures did not, which could be impacted on by numerous confounding factors. Toxicity of chemotherapy was manageable with no post-operative deaths and only a 4% surgical complication rate observed in the chemotherapy group. Neoadjuvant chemotherapy and targeted agents did not cause any evidence of pulmonary toxicity, either radiologically or histologically. FDG-PET scanning, also specifically evaluated for the first time in our series, provides higher concordance with the number of histologically proven metastases present than CT when PET-avid lesions are present (72% versus 61%).

Published series of pulmonary metastasectomy have included variable numbers of patients who received systemic peri-operative treatment but detailed outcomes of these subgroups have not been specifically reported. In a systematic review of 11 retrospective studies including 1307 patients [3], 8 of the 11 studies reported the use of neoadjuvant or adjuvant chemotherapy but discussion of toxicity, response, details of the regimen were consistently lacking. Two reports within the review, and two further published series have evaluated peri-operative chemotherapy as a prognostic factor. Saito et al [18]. (n = 165) and Lee et al [7]. (n = 59) both found administration of peri-operative chemotherapy to be non-significant as a prognosticator in multivariate analysis. In a series of 30 patients, 5-year OS appeared higher in patients receiving post-operative chemotherapy (83.5 versus 57.1%) yet 5-year disease-free survival was similar (45.8% versus 46.9%), though in this small cohort, neither was statistically significant (p = 0.397 and 0.754 respectively) [19]. A larger study of 315 patients by Kanemitsu et al [20]. reported 27.2% receiving ‘adjuvant chemoradiotherapy’. Univariate analysis demonstrated a significant benefit in undergoing adjuvant therapy (Hazard ratio for death at 3 years 0.69, 95% CI 0.49-0.98; p = 0.037) with a non-significant trend towards reduction in death at 3 years in multivariate analysis (HR 0.71, 95%CI 0.49-1.03; p = 0.068).

The 5-year OS of 72% in our cohort was higher than other published series. The recent systematic review reported a median 5-year OS of only 39.6% (range: 24-56%) for R0 resected patients [21]. Our OS was promising and the low morbidity and mortality we reported supports the use of pulmonary resection and peri-operative chemotherapy as a treatment modality. With our 5-year RFS of 31% after initial resection and 3 additional patients being rendered disease-free after subsequent resections, pulmonary resection appears beneficial in this selected group, and re-resection in a small number of patients is also a feasible option.

Neoadjuvant chemotherapy also has the advantage of assessing in vivo tumour response and overall course of the disease as well as potentially downsizing lesions. Whilst we acknowledge that the majority of lung lesions in this study are not evaluable by RECIST due to small size, a reduction in radiological tumour size was documented in 62% of resections where neoadjuvant chemotherapy was administered, with an additional 30% having stable disease. Indeed, although the presence of necrosis and, to a lesser extent, fibrosis are features seen in metastatic colorectal carcinoma, the extent of these changes being up to 80% likely reflects partial response to chemotherapy prior to surgery in some cases. Progression was seen in only a small minority (8%) during neoadjuvant therapy. Obviously, due to selection bias, we recognise that the cohort of patients who progressed, becoming unresectable, were not captured in this study nor did any lesions require downsizing prior to consideration of resection. However, this response was similar to published response rates in metastatic CRC of 36-66% [22-24].

DFI following curative resection of the primary has been described as a possible prognostic determinant. In the CS group, DFI was shorter than in the S group, suggesting that those with a shorter DFI are being treated more aggressively. Despite the slightly shorter DFI in this group (20.5 months versus 27.5 months, p = 0.277), the absolute 5-year OS was higher (74% vs. 68%).

This study demonstrates that chemotherapy toxicity appears manageable in this population. Pfannschmidt’s 2010 systematic review reported a 0–2.4% post-operative mortality, consistent with our observation of no surgery-related deaths [3]. Acute surgical complications were observed less in the chemotherapy group in our study (3 events in 68 resections versus 5 events in 16 resections for surgery alone). Neoadjuvant chemotherapy was better tolerated than adjuvant chemotherapy with fewer recorded toxicities (reported in 27% versus 54% of patients). This difference in toxicity pre- and post-surgery is consistent with reports in operable rectal and gastric cancers where neoadjuvant and adjuvant chemotherapy have been evaluated [25-27]. Of interest, in patients who received targeted therapy, only 2 complications occurred. A patient receiving neoadjuvant sunitinib plus FOLFIRI (within the context of a phase I dose-escalation study) developed grade 4 neutropenia pre-operatively and a patient receiving FOLFIRI chemotherapy plus cetuximab had a prolonged air-leak. None of the toxicities or surgical complications occurred in the 6 patients receiving chemotherapy plus bevacizumab with the median interval between end of neoadjuvant treatment and surgery of 37 days, despite the widely published wound-healing, bleeding and thromboembolic complications known to occur with bevacizumab. There was no radiological evidence of chemotherapy-related pulmonary toxicity and histological evaluation of the resected tissue demonstrated no evidence of toxic changes to non-tumour lung parenchyma. This is reassuring, and certainly in contrast to that of neoadjuvant chemotherapy in the setting of liver resection, where it is well documented that irinotecan can cause steatohepatitis and oxaliplatin is associated with sinusoidal obstruction syndrome [28]. There are currently no data on chemotherapy-induced lung toxicity seen histologically within the context of resectable CRC pulmonary metastases and our report represents first preliminary evidence demonstrating no significant histological damage is detected following CRC chemotherapy.

CT is not highly accurate in differentiating malignant from benign pulmonary nodules, hence many benign lesions are removed in pulmonary metastasectomy. FDG-PET is used routinely to confirm a single metastatic site of disease prior to resection however may assist in further delineating malignant involvement of pulmonary lesions. Concordance of FDG-PET with histological involvement of pulmonary nodules has not previously been assessed. In patients undergoing neoadjuvant chemotherapy, the timing of preoperative PET varied in our cohort, yet administration of chemotherapy did not impact on FDG-avidity of the lesions. In PET-positive patients, we observed a higher concordance between number of lesions with positive histology and PET than CT. These concordance rates were exploratory and hence only described in absolute values, the limited sample size precluded any formal statistical testing. With further evaluation, this may have important consequences on the decision of which lesions to resect at surgery in addition to excluding extrapulmonary disease.

The most important limitation of our study was the selectivity of our approach. Included patients needed to have resectable pulmonary metastases, physical fitness for lung resection and no progression during neoadjuvant chemotherapy to the extent of being denied surgery. In patients who had significant co-morbidities, radiofrequency ablation might have been utilised as an alternative. High usage of PET scanning in our study ensured that patients with extra-thoracic non-hepatic metastases were excluded from pulmonary resection. As an indication, our institution commenced 670 patients on first line chemotherapy for metastatic CRC between 2000 and 2008 (equivalent of about 80 patients per year) [29].

## Conclusions

To our knowledge, this is the largest study of patients dedicated to evaluating peri-operative chemotherapy and targeted therapy in the setting of pulmonary resection for CRC metastases. The role of pulmonary metastectomy has not been validated in a prospective randomised trial to date however based on published data, has been standard practice for selected patients at our intuitions for more than a decade. The inclusion of perioperative chemotherapy in the management paradigm for these patients has not been associated with additional specific pulmonary toxicity and a promising relapse-free and overall survival was observed.

## Competing interests

No authors have declared any conflict of interests.

## Authors’ contributions

EH: Study design, data collection, data analysis, manuscript preparation, manuscript review. GL: Study design, data collection, data analysis, manuscript preparation, manuscript review. DC: Study design, manuscript preparation, manuscript review. AN: Study design, data collection, data analysis, manuscript preparation, manuscript review. KW: Data collection. YB: study design and statistical analysis. GR: Data collection. SR: Study design, manuscript preparation, manuscript review. IC: Study design, manuscript preparation, manuscript review. All authors read and approved the final manuscript.

## Pre-publication history

The pre-publication history for this paper can be accessed here:

http://www.biomedcentral.com/1471-2407/12/326/prepub
